# Identification of Rapeseed (*Brassica napus* L.) Plant Height-Associated QTL Using BSA-seq and RNA-seq

**DOI:** 10.3390/ijms25189875

**Published:** 2024-09-12

**Authors:** Jichun Xia, Lanlan Zhan, Jiaying Zhang, Wenhui Song, Xinfu Xu

**Affiliations:** 1 College of Agronomy and Biotechnology, Southwest University, Beibei, Chongqing 400715, China; xjc199802@email.swu.edu.cn (J.X.); zhanll@email.swu.edu.cn (L.Z.); zhangjy@email.swu.edu.cn (J.Z.); songwh@email.swu.edu.cn (W.S.); 2 Academy of Agricultural Sciences, Southwest University, Beibei, Chongqing 400715, China

**Keywords:** *Brassica napus* L., plant height, semi-dwarf, BSA-seq, RNA-seq

## Abstract

Plant height (PH) is a critical agronomic trait in *Brassica napus*, significantly impacting yield. Consequently, identifying genes associated with plant height is a pivotal objective in oilseed rape breeding. This study employed a combination of bulk segregant analysis sequencing (BSA-seq) and RNA sequencing (RNA-seq) for analysis. A novel quantitative trait locus (QTL), *qPH_C02*, was identified between 63,989,634 and 64,945,122 bp on chromosome C02, from which eight candidate genes were screened. The Gene Ontology (GO) analysis revealed enrichment in peroxisomes, while the Kyoto Encyclopedia of Genes and Genomes (KEGG) analysis indicated enrichment in the oxidative phosphorylation (OP) pathway. It is hypothesized that the observed differences in plant height and silique length may be attributed to the regulation of peroxidase activity in the OP pathway, which in turn alters plant energy metabolism and controls nutrient uptake. Subsequently, we will further test this hypothesis. The results of this study will contribute to our understanding of the genetic basis for differences in plant height and provide a foundation for the selection and breeding of *Brassica napus* varieties with desired plant shapes.

## 1. Introduction

Rapeseed (*Brassica napus*, AACC, 2*n* = 38) is one of the world’s top three oil crops. Plant height (PH) is an important agronomic trait in rapeseed, directly affecting yield via the number of siliques per plant and the number of effective branches [1]. Additionally, PH correlates significantly with lodging in rapeseed [2]. Varietal use of hybrid rapeseed increased PH by ~20 cm, amplifying lodging risk and hindering mechanical harvest [3]. Consequently, reducing PH is crucial for mitigating lodging risk, with the development of dwarfing resources playing a pivotal role in rapeseed dwarf breeding. However, dwarfing resources in rapeseed are limited, and dwarfing mechanism research is insufficient, greatly hindering dwarfing goals in rapeseed breeding [4,5,6]. Hence, exploring potential dwarfing resources with valuable applications holds significant implications for rapeseed dwarf breeding.

Crop dwarfing is often associated with plant hormone biosynthesis or signal transduction pathways, as evidenced by the modulating of PH through the regulation of gibberellic acid (GA) synthesis, metabolism, and signal transduction during the “Green Revolution” [7,8]. For instance, rice *sd1* and wheat *Rht* genes induce plant dwarfism by suppressing DELLA protein accumulation, consequently enhancing lodging resistance and grain yield [9]. The allelic gene *Rht24* also exhibits substantial breeding potential by enhancing GA metabolic enzyme TaGA2ox-A9 expression, reducing PH without affecting yield, and significantly improving nitrogen use efficiency and photosynthetic rate [10,11]. Brassinosteroids (BRs) are widely distributed in plant tissues and participate in numerous biological processes involved in plant growth and development [12,13]. BR-insensitive (BRI) mutants (e.g., *bri1*) display dwarfism, reduced cell elongation rate, dark green and thickened leaves, diminished apical dominance, delayed flowering and senescence, altered vascular patterns, and male sterility [14]. When driven by the BRI1 promoter, BRL1 and BRL3 can bind with BRs and restore the phenotypes of BRI1 mutants [15,16]. Auxin, an important regulator of plant development, primarily originates from the apical tips of stems and young leaves and is involved in regulating cell division, elongation, and differentiation [17,18]. In regulating cell elongation, auxin interacts with gibberellins. Both auxin and gibberellins promote microtubule reorientation from random to transverse, inhibit peroxidase activity, and regulate plant cell elongation growth by stimulating cell wall extension, promoting main stem elongation, and suppressing lateral bud growth to adjust plant height [19]. The dominant dwarfing gene ds-4 encodes an Aux/IAA protein that regulates PH in *Brassica napus* by acting as a deterrent in growth hormone signaling [20].

Non-hormonal pathways also exert influences on PH and are closely associated with dynamic changes in the plant cell wall [21]. The extensibility of the cell wall controls the growth rate of plant cells, particularly cell expansion [22]. Expansins, a class of cell wall-loosening proteins, are essential in many key developmental processes, facilitating cell enlargement [23]. In addition to expansins, xyloglucan endotransglucosylase/hydrolases (XTHs) are also implicated in regulating cell wall loosening [24]. XTHs catalyze the endohydrolysis of xyloglucan polymers and subsequently reassemble them, enabling the movement of cellulose microfibrils with cell expansion or elongation [25]. Endo-1,4-beta-glucanase (Egase) is considered to be an enzyme that modifies the hemicellulosic network and participates in processes that require cell wall weakening, including cell elongation, organ abscission, and fruit softening [26].

Plant height in rapeseed is a quantitative trait controlled by multiple genes, which is greatly affected by the environment and shows continuous variation. Using different genetic materials, researchers have identified plant height QTLs on all 19 chromosomes of kale-type oilseed rape, with individual QTL contribution rates ranging from 0.7% to 54.59%, laying the foundation for the genetic improvement of plant height [2,27,28,29,30,31,32,33,34]. Zhao detected 18 plant height-related QTLs on chromosomes A02, A03, A07, A10, C01, C03, C04, C06, and C09 using a DH (doubled haploid) population [29]; Luo genotyped BnaTNDH (a doubled-haploid population) and reanalyzed previous phenotypes, detecting 80 plant-height-related QTLs on all chromosomes except C01 [30]. He detected nine plant height-related QTLs in the A02, A09, C01, C02, and C06 clusters using one DH population and one permanent F2 population [1]. Although many PH-affected genes have been cloned and have shown functional value [7], the genetic basis of dwarfing resources remains limited [7], and studies using semi-dwarfing genes to alleviate crop collapse are still scarce [9]. Further in-depth or refined QTL studies on oilseed rape plant height are still needed. In this study, we crossed two materials with plant height differences, 21Y490 (with high plant height) and 21Y689 (with semi-dwarf plant height), to obtain an F2 segregating population and employed BSA-seq, RNA-seq sequencing, and QTL mapping to screen for height-related QTLs. By identifying height-regulating loci with stable and specific expression during critical growth stages, we aim to provide resources and a theoretical basis for breeding high-yielding, superior-quality semi-dwarf rapeseed varieties suitable for mechanical harvesting.

## 2. Results

### 2.1. Analysis of Height Variation in the F2

**Figure 1:**
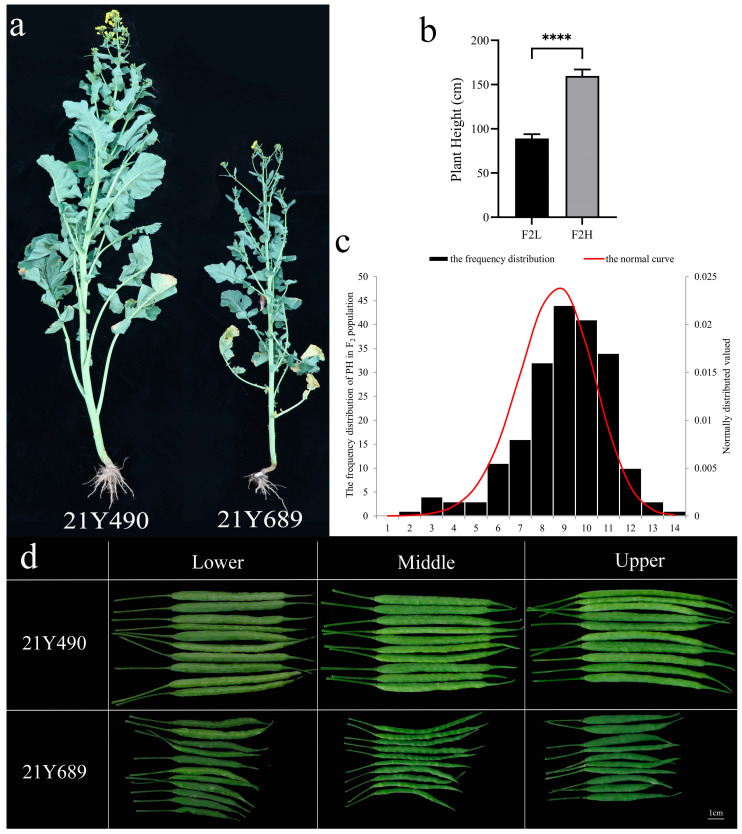
Heights of parental lines at the early flowering stage (**a**), mixed F2 population (**b**), F2 population frequency distribution (**c**), silique length of different parts (**d**). Bars represent means ± SE (*n* = 30). Asterisks indicate statistically significant differences from Student’s *t*-test (**** *p* < 0.0001).

We examined the plant heights of the two parents (21Y689 and 21Y490) and the 203 F2 plants at different times, finding obvious differences in plant heights from the early flowering stage. The average plant heights of the parents were 145 cm and 203 cm, respectively (Figure 1a), and those of the F2 populations ranged from 85 cm to 179 cm, with an average of 134.32 ± 19.10 cm (Figure 1b). The absolute values of skewness and kurtosis were less than one, and the coefficient of variation (0.14) was relatively large and normally distributed (Figure 1c). The Kolmogorov–Smirnov test on the data showed *p* > 0.05, confirming normality (Table S1), a result consistent with the genetic characterization of quantitative traits. Moreover, we observed that the silique length of the taller parents was significantly longer than those of the semi-dwarf parents (Figure 1d).

### 2.2. Paraffin Section Analysis

**Figure 2:**
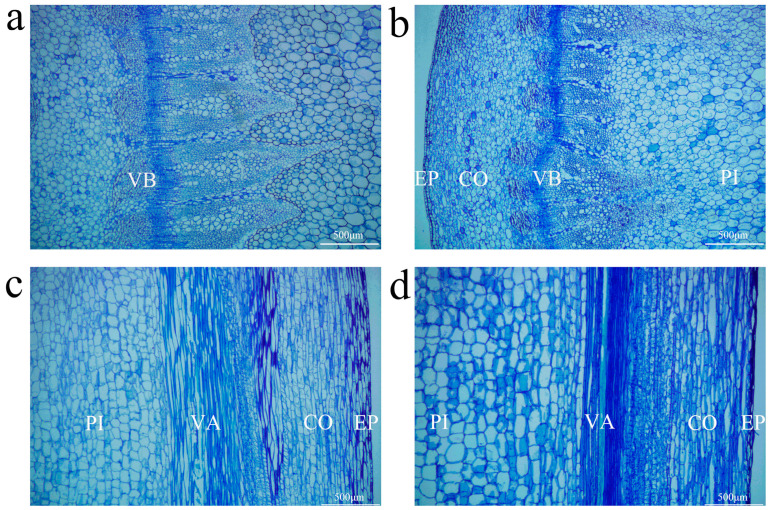
Culm slices of *Brassica napus* parent material. Cross section of tall parent stem (**a**); cross section of stalk of semi-dwarf parent (**b**); longitudinal section of the stalk of tall parent (**c**); longitudinal section of stalk of semi-dwarf parent (**d**). EP—epidermis; CO—cortex; VB—vascular bundle; PI—pith part; VA—vascular tissue, xylem, and phloem.

Changes in the cellular morphology of the parental strain were analyzed through paraffin section examination. The cross sectional results revealed that both the semi-dwarf rapeseed variety (21Y689) and the tall rapeseed variety (21Y490) exhibited only one layer of epidermal cells arranged in a highly compact, relatively regular manner. The epidermal cells of 21Y689 had smaller intercellular spaces and a tighter arrangement. The vascular bundles were tightly wrapped around each other within a relatively smaller area, and the arrangement of xylem cells was more compact compared to that of 21Y490. In contrast, the longitudinal section analysis showed that the cellular arrangement in the stem of 21Y689 was even denser and more compact, with a significantly lower cell count compared to 21Y490. Furthermore, the semi-dwarf material 21Y689 exhibited more regular cell shapes (Figure 2).

### 2.3. Bulked Segregant Analysis (BSA)

**Figure 3:**
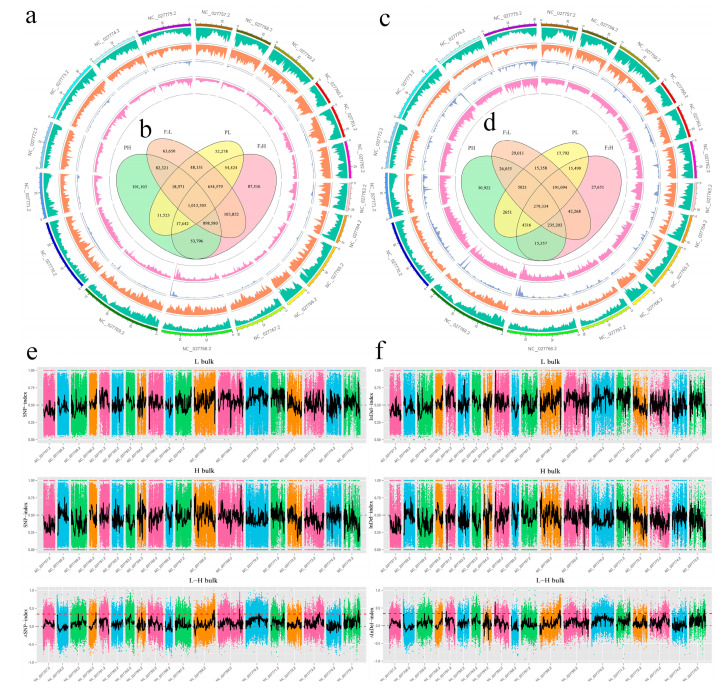
SNP and InDel statistics among samples and their distribution on chromosomes. Genome-wide visualization of SNPs (**a**), Venn diagram of SNP differences among samples (**b**), genome-wide visualization of InDels (**c**), Venn diagram of InDel differences among samples (**d**), distribution of SNPs on chromosomes (**e**), and distribution of InDels on chromosomes (**f**). The first circle in the figure represents chromosome coordinates, the second one indicates gene distribution, the third one refers to the density distribution of SNPs and InDels, and the fourth one represents the ED value distribution of SNPs and InDels from outside to inside.

Whole-genome resequencing of two parental lines yielded 147.47 Gbp of clean reads after data filtering, with a Q30 score of 93.80%. The average alignment efficiency with the reference genome was 98.86%, with an average coverage depth of 25.00X and a genome coverage of 95.63% (Table 1). A total of 1,871,954 SNPs were identified between parental lines, including 101,355 non-synonymous SNPs. In pooled samples, 699,960 SNPs were identified, with 33,193 being non-synonymous (Figure 3a,b). Furthermore, 531,787 and 226,245 InDels were identified between parental lines and pooled samples, respectively (Figure 3c,d). Subsequently, SNP/InDel sites were subjected to association analysis using the ED and index methods (Figure 3e,f). Regions identified by both methods were intersected to obtain candidate genes, resulting in 901 and 401 candidates for SNP and InDel analysis, respectively. The intersection of SNP and InDel-associated regions yielded three candidate chromosomal segments, namely NC_027768.2 (BnaC02), NC_027769.2 (BnaC03), and NC_027775.2 (BnaC09), totaling 4.06 Mb with 387 genes within intervals, including 113 non-synonymous and 21 frameshift mutation genes. Among candidate regions, BnaC02 showed a clear, continuous peak, making it the primary focus for further candidate gene selection (Table 2).

### 2.4. Narrowing Down Candidate Regions

**Figure 4:**
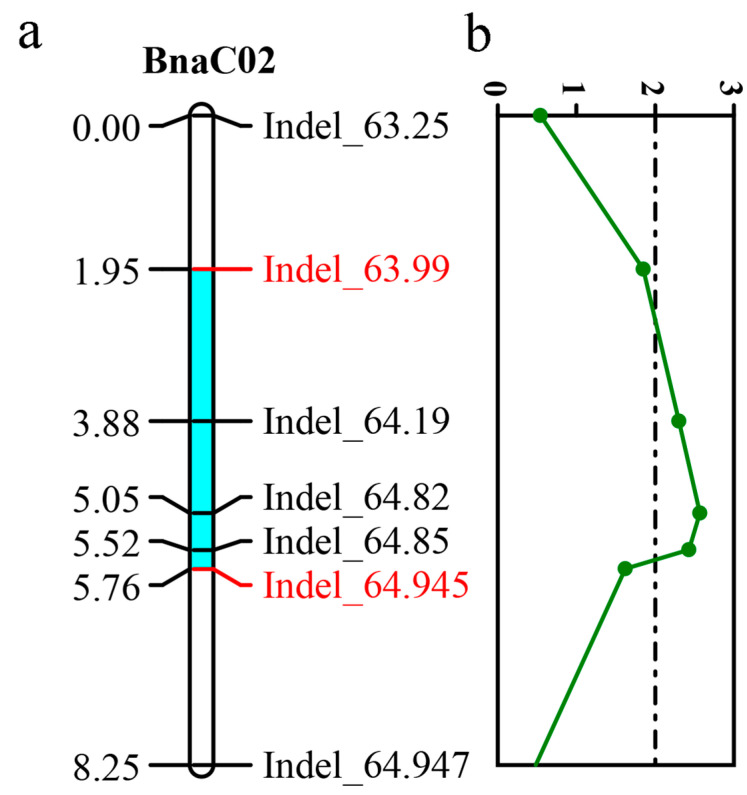
Construction of the genetic map for *qPH_C02* (**a**). Genetic mapping of candidate intervals on BnaC02; blue regions indicate confidence intervals for *qPH_C02* (**b**). InDel markers correspond to LOD values, with LOD = 2.0 as the threshold.

Within the candidate region on chromosome C02, 64 InDel primers were designed. Polymorphism screening was conducted using tall-stemmed and short-stemmed parental material on these InDel primers, yielding seven pairs of polymorphic primers (Table S2), spanning a total genetic map length of 8.25 cM. We localized a plant height QTL (*qPH_C02*) between Indel_63.99 and Indel_64.945, with a genetic distance of 3.81 cM and a physical distance of 0.96 Mb (Figure 4). The LOD value of *qPH_C02* was 2.57, and the additive effect was 9.9, explaining 5.2% of the phenotypic variance. There are 69 genes within this region, of which 24 possess non-synonymous mutations.

### 2.5. Comparative RNA-seq for PH

**Figure 5:**
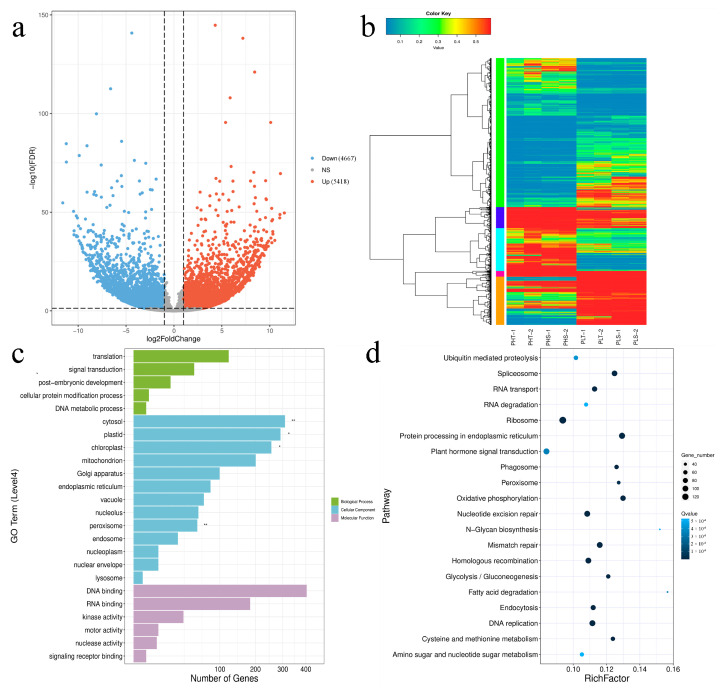
Differentially expressed gene analysis. (**a**) Volcano plot of differential expression analysis; (**b**) clustering map of differentially expressed genes; (**c**) bar chart of GO function enrichment, with the *x*-axis representing the number of different genes annotated to GO term (* indicates Q-value < 0.05; ** indicates Q-value < 0.01) and *y*-axis representing GO term. Different colors represent three GO subclasses BP, CC, MF. (**d**) Scatter plot of KEGG pathway enrichment, with the *x*-axis representing the ratio of the number of different genes annotated to the KEGG pathway to the total number of different genes’ Rich factors—the larger the Rich factor, the higher the enrichment. *y*-axis represents the KEGG pathway. The size of the dot represents the number of genes added to the KEGG pathway. The color represents the significance of enrichment—the deeper the color, the greater the significance.

**Figure 6:**
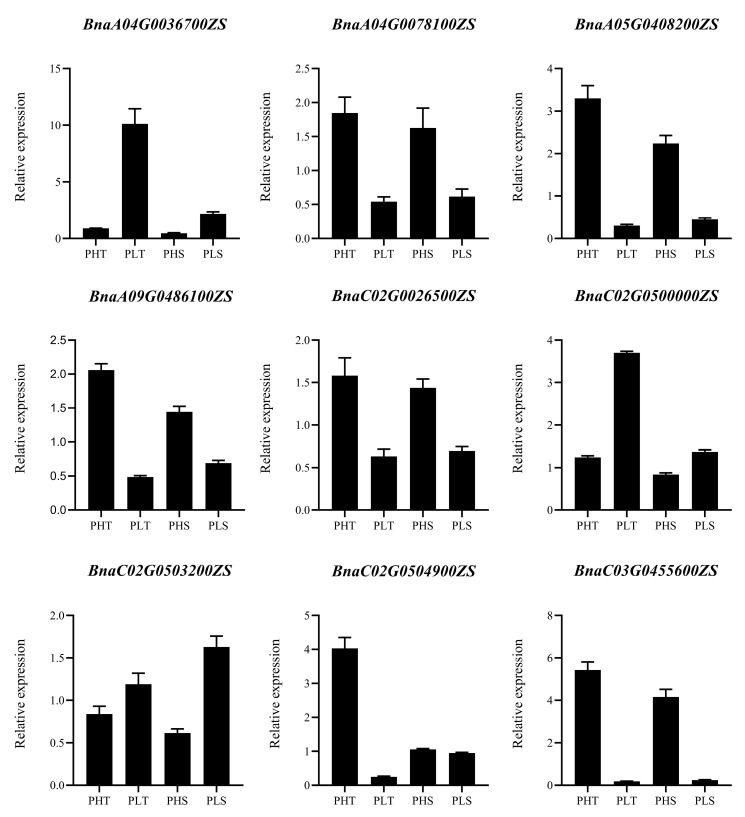
Verification of the transcriptome results by qRT–PCR. *BnActin7* was used as an internal control.

To explore the regulatory mechanisms underlying plant height differences and to fully characterize differentially expressed genes (DEGs) between two parents, we performed transcriptome analysis using stem tips and the upper stem section (10 cm from the tip) at the early flowering stage. After filtering raw data, an average of 8.12 Gb clean data was obtained for each sample with an average Q30 value of 94.2%, indicating high-quality RNA-seq data (Table S3). Amongst parents and progeny, 10,085 differentially expressed genes (DEGs) were identified, with 5418 upregulated and 4667 downregulated (Figure 5a), implying their involvement in height development regulatory networks. Nine DEGs (BnaA09G0486100ZS, BnaA04G0078100ZS, BnaA04G0036700ZS, BnaC03G0455600ZS, BnaC02G0026500ZS, BnaA05G0408200ZS, BnaC02G0503200ZS, BnaC02G0500000ZS, BnaC02G0504900ZS) were selected from the RNA-seq data for qRT-PCR validation; the results showed that the relative expression levels of DEGs detected by qRT-PCR were mainly consistent with RNA-seq data (Table S4, Figure 6). Hierarchical clustering analysis revealed similar gene expression patterns within the same group (Figure 5b). GO enrichment was carried out to investigate biological processes of DEGs during the PH elongation stage (Figure 5c). A bar chart was created to show the 30 most significant pathways. Analysis identified concentrations in “DNA binding”, “cytosol”, “plastid”, “chloroplast”, “RNA binding”, and “translation”. Upregulated DEGs were primarily enriched in peroxisome and cytosol functions, while downregulated DEGs mainly concentrated on peroxisome, plastid, cytosol, and chloroplast functions (Figure S1a,b). From KEGG enrichment analysis results, a bubble chart was created with the 20 most significant pathways. Differentially expressed genes were primarily concentrated in the ribosome pathway and the protein processing in the endoplasmic reticulum pathway (Figure 5d). Upregulated DEGs were mainly concentrated in the sulfur metabolism pathway, while downregulated genes were primarily concentrated in the oxidative phosphorylation pathway (Figure S1c,d).

## 3. Discussion

### 3.1. qPH_C02 Is a New PH QTL

PH, as a key component of crop typology, critically affects crop yield and lodging resistance alongside other agronomic traits. Using semi-dwarf resources to moderately reduce height enhances yield, lodging resistance, and harvestability in oilseed rape, revealing a major breeding strategy [35,36,37,38]. Therefore, identifying QTLs associated with PH holds potential for discovering height-related genes and breeding high-yielding oilseed rape varieties. PH in *Brassica napus* is a quantitative trait influenced by both genetic and environmental factors [27,28]. To date, QTLs associated with PH have been identified in all 19 chromosomes in *Brassica napus*. There are abundant molecular markers and functional genes closely linked to PH or dwarf traits [2,29,30,31,32,33,34]. We located *qPH_C02* on chromosome C02 between 63,989,634 and 64,945,122 bp, spanning 955 kb. Although previous studies identified height-related regions on BnaC02 [30,39], none of these regions overlapped with *qPH_C02*, suggesting that it may represent novel loci regulating PH development.

### 3.2. Oxidative Phosphorylation Regulates PH

Gene Ontology (GO) enrichment analysis revealed that differentially expressed genes were significantly enriched in peroxisomes (Figure 5c). Peroxisomes play vital metabolic roles in plants, including photorespiration, fatty acid and amino acid metabolism, hormone metabolism, reactive oxygen species metabolism, coenzyme synthesis, and purine metabolism [40]. They are closely linked to crop yield and resistance, participating broadly in plant physiological processes. A plethora of studies suggest that cell growth, cell wall loosening, lignification, biotic and abiotic stress responses, plant growth, maturation, and senescence are all significantly related to peroxisomes [41,42,43,44]. In the KEGG database, the downregulated genes were mainly enriched in the oxidative phosphorylation pathway. Paraffin tissue sectioning showed well-developed xylem and phloem in both short and tall plants, ensuring the necessary transport of water, inorganic salts, and organic substances during growth. However, tall oilseed rape has larger vascular bundles that facilitate nutrient transport and accumulation (Figure 2). Therefore, we speculate that these findings may be due to the oxidative phosphorylation pathway, which regulates peroxidase activity and thereby alters the plant’s energy metabolism and controls its nutrient uptake, which in turn results in differences in PH and angiosperms.

## 4. Materials and Methods

### 4.1. Plant Materials

Two rapeseed varieties (material 21Y490 and material 21Y689) were used in this study. The line 21Y490 (with high plant height) is an inbred line that has been maintained in our laboratory for several years, while 21Y689 (with semi-dwarf plant height) is a naturally occurring variant selected from the field. In March 2021, 21Y689 was crossed with 21Y490 as the female parent, and the F1 hybrid was self-pollinated through summer propagation to generate F2 seeds. In the fall of 2021, all materials were grown at Southwest University of Beibei, Chongqing, China (29°45′N, 106°22′E, 238.57 m). Seedlings were transplanted with 10 plants per row, 40 cm between rows, and 20 cm between plants, following conventional production methods and field management.

### 4.2. Cytological Analysis of Rapeseed Stem Tissues

At the early flowering stage, two plants of each parent material were selected. Stem segments 10 cm below the plant apex were excised and fixed in FAA fixative solution (50% ethanol, 0.9 M ice-cold acetic acid, and 3.7% formaldehyde) by vacuum infiltration. The fixed samples were stored at 4 °C. Standard paraffin sectioning techniques were used, starting with dehydration in a series of ethanol solutions (70%, 85%, 95%, and 100%) before clearing the samples with xylene, embedding them in paraffin, and cutting them into 6–8 μm thick sections. The sections were then stained with toluidine blue solution, mounted with neutral gum, and observed and photographed under a fluorescent microscope (Nikon E600, Nikon Instruments Inc., Tokyo, Japan).

### 4.3. BSA-seq Analysis

Leaf samples were collected and stored at −80 °C. Based on phenotypic identification of the F2 segregating population for PH, 30 extremely tall and 30 dwarf plants were selected. Genomic DNA was extracted using the cetyltrimethylammonium bromide (CTAB) method [45]. The concentration and quality of the extracted DNA were determined using a NanoDrop 2000 spectrophotometer (NanoDrop, Wilmington, DE, USA). The tall and dwarf plant pools (F2H, F2L) were constructed using DNA samples of selected plants. The pools along with the parental DNA samples were sent to Beijing Biomarker Technologies Co., Ltd. (Beijing, China) for library construction and whole-genome resequencing on the Illumina HiSeq platform. The reference genome sequence was downloaded from the National Center for Biotechnology Information (*Brassica napus* genome assembly Bra_napus_v2.0-NCBI-NLM (https://www.nih.gov/)). The sequencing quality was assessed based on the quality and type of bases, and low-quality reads (including reads with adapters, N ratio > 10%, and quality value (Q) ≤ 10) were removed to obtain clean reads. The clean reads were aligned to the reference genome using the Burrows–Wheeler Aligner (BWA) software (version 0.7.10) [46]. SNP and small InDel detections were performed using the Genome Analysis ToolKit (GATK, version 3.7) [47] package. Detected variants were annotated using the SnpEff software (version 3.0) [48]. The association regions were analyzed using both the Euclidean Distance (ED) algorithm and the SNP-index method [49]. The intersection of the results obtained from both methods was used as the candidate region. The coding genes within the candidate region were annotated using the NR [50], Swiss-Prot [51], GO [52], KEGG [53], and COG [54] databases through the BLAST [55] software (version 2.14.0).

### 4.4. Delimitation of Candidate Region

Genomic DNA was extracted from the young leaves of the parents and the F2 population at the budding stage. InDel markers were screened based on the results of BSA-seq, and the Primer3 software (version 0.4.0) was used to design primers. Screening revealed polymorphic markers in the two parents. These markers were then validated and analyzed in the F2 population. The range of information obtained from validating the progeny population was visualized using the Joinmap 4.0 [56] and MapQTL 6.0 [57] software to further narrow down the candidate region.

### 4.5. Transcriptome and Quantitative Real-Time PCR (qRT-PCR) Analysis

Stem tip and upper stem (10 cm from the tip) samples were collected from two parents, 21Y490 and 21Y689, at the early flowering stage. Two biological replicates were set for each sample, totaling eight samples. The total RNA was extracted using the SteadyPure Plant RNA Extraction Kit (Aidlab Biotechnologies Co., Ltd., Beijing, China) and assessed for concentration and quality via a NanoDrop 1000 spectrophotometer and agarose gel electrophoresis. Qualified samples were sent to Genoseq Technology Co., Ltd. (Wuhan, China) for sequencing. Clean reads were mapped to the *Brassica napus* reference genome (Bra_napus_v2.0-NCBI-NLM (https://www.nih.gov/)) using HISAT2 v2.1.0 [58]. Gene expression levels were quantified using RSEM v1.2.31 [59], and expression values (TPM) were calculated for all genes in each sample. The data were normalized via the estimateSizeFactors function in the DESeq R package [60], and *p*-values and fold-change values for differential expression were calculated using the nbinomTest function. Genes with a *p*-value < 0.05 and fold-change > 2 were considered differentially expressed, and annotation was performed using NR [50], GO [52], KEGG [53], COG [54], and Swiss-Prot [51] databases.

To validate sequencing accuracy, 9 differentially expressed genes (BnaA09G0486100ZS, BnaA04G0078100ZS, BnaA04G0036700ZS, BnaC03G0455600ZS, BnaC02G0026500ZS, BnaA05G0408200ZS, BnaC02G0503200ZS, BnaC02G0500000ZS, BnaC02G0504900ZS) were randomly selected for qRT-PCR validation. Using 8 samples of previously extracted RNA, complementary DNA was synthesized from equal amounts of RNA using the PrimeScript™ RT reagent kit and then diluted 10 times for qRT–PCR, in accordance with the manufacturer’s instructions (TaKaRa Biotechnology, Dalian, China). We downloaded the gene CDS sequences based on their accession numbers, designed qRT–PCR primers using Primer3 (Version 0.4.0), and checked their specificity in NCBI. Primers were synthesized by Beijing Qingke Biosciences Co., Ltd. (Beijing, China). The sequences are provided in Table S4. qRT-PCR reactions were performed using the ChamQ Universal SYBR qPCR Master Mix kit (Vazyme Biotech, Nanjing, China) on a CFX96 real-time PCR instrument (Bio-Rad Laboratories, Hercules, CA, USA). BnActin7 was used as an internal control, and data were analyzed via the 2^−ΔΔCt^ method with the Bio-Rad CFX Manager 3.0 software and GraphPad Prism 9.0 [61].

## 5. Conclusions

In this study, BSA-seq was used to design InDel primers in the candidate interval to precisely map the *qPH_C02* locus between 63,989,634 and 64,945,122 bp on chromosome C02. RNA-seq analysis of the extreme parents revealed that GO terms were mainly enriched in peroxides and KEGG pathways were enriched in the oxidative phosphorylation pathway. We hypothesize that the oxidative phosphorylation pathway may regulate peroxisome activity and alter energy metabolism and nutrient uptake in plants, resulting in differences in PH and silique length. Subsequently, we will further test this hypothesis. These results will lay the foundation for elucidating the molecular mechanisms underlying the differences in PH.

## Figures and Tables

**Table 1:** Sample sequencing data and comparison results with reference genomes.

Sample	Clean_Reads	Clean_Base	Q20 (%)	Q30 (%)	Mapped (%)	Ave_Depth (X)
F2H	160,784,140	48,047,489,686	97.73	93.86	99.23	32
F2L	198,133,337	59,214,890,472	97.47	93.29	92.73	39
PH	62,044,117	18,544,946,240	98.17	94.58	98.80	13
PL	72,504,684	21,664,185,030	97.55	93.47	92.20	16

Clean_Reads—post-filtered reads; Clean_Bases/filtered base number—clean reads times the length of the sequence; Q20 (%)—the percentage of bases with a mass value greater than or equal to 20; Q30 (%)—the percentage of bases with a mass value greater than or equal to 30; mapped (%)—the percentage of clean reads that are located in the reference genome is the percentage of all clean reads; ave depth—average depth of sample coverage; F2H—30 extremely tall plants in the F2 population; F2L—30 dwarf plants in the F2 population; PH—high plant height in the parents; PL—semi-dwarf plant height in the parents.

**Table 2:** Statistical table of BSA-associated sequencing information.

Chromosome_ID	Start	End	Size (Mb)	Gene Number
NC_027768.2 (BnaC02)	63,260,000	65,420,000	2.16	182
NC_027769.2 (BnaC03)	68,760,000	69,680,000	0.92	76
NC_027775.2 (BnaC09)	47,080,000	48,060,000	0.98	129
Total	-	-	-	387

Chromosome_ID—the chromosome number; start— the start position of the associated region; end— the end position of the associated region; size— the size of the associated region in megabases (Mb); Gene_Number— the number of genes within the associated region.

## Data Availability

Data used in this study are presented in the Supplementary Materials.

## Supplementary Materials

The following supporting information can be downloaded at https://www.mdpi.com/article/10.3390/ijms25189875/s1.
