# Supplementary material for: Identification of Rapeseed (Brassica napus L.) Plant Height-Associated QTL Using BSA-seq and RNA-seq
Source: Int J Mol Sci. 2024 Sep 12;25(18):9875. doi: 10.3390/ijms25189875 (PMC11432562; doi:10.3390/ijms25189875)
Supplement: Supplementary file 1 [file ijms-25-09875-s001.zip › Figure S1.pdf]

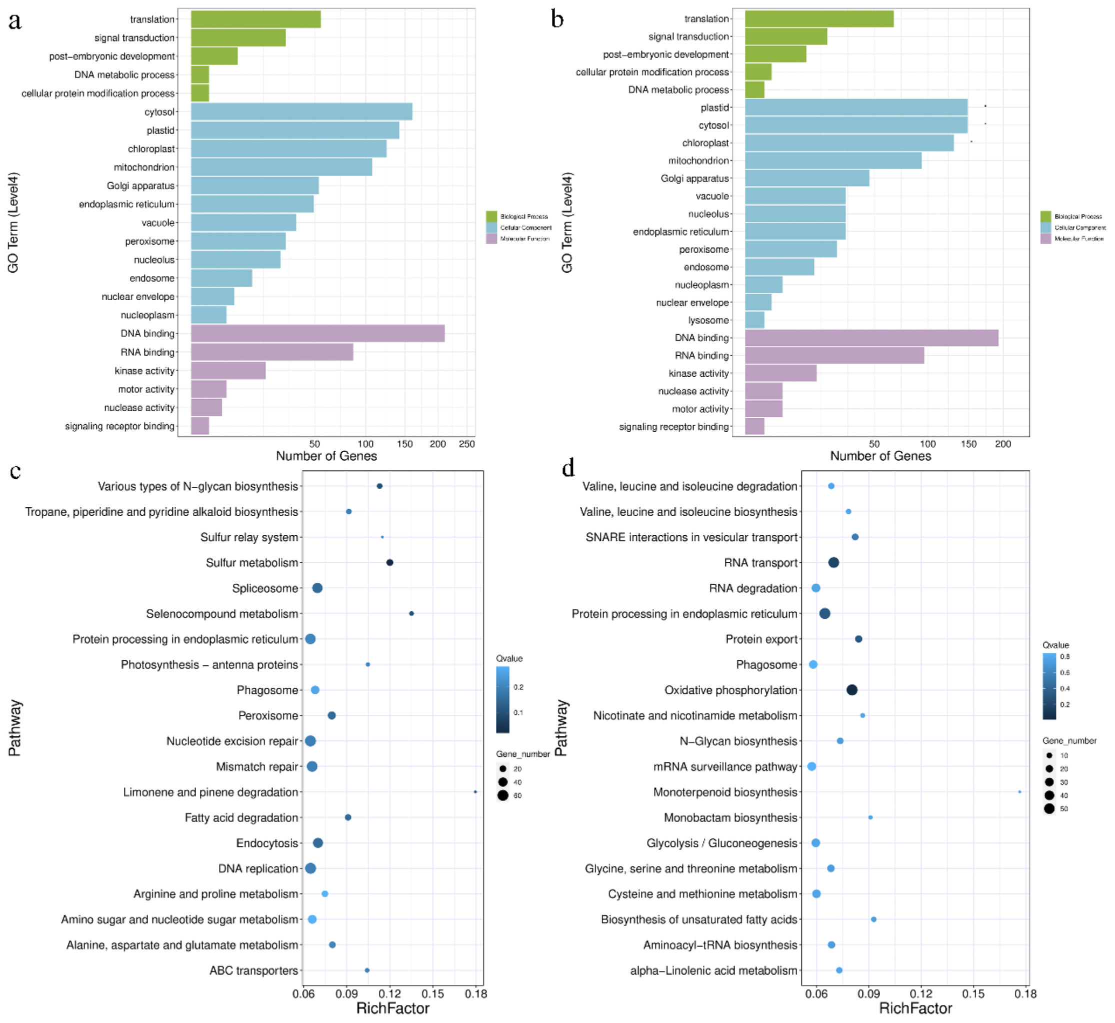

Figure S1. (**a**) Upregulated genes' GO enrichment analysis; (**b**) downregulated genes GO enrichment analysis, with the x-axis representing the number of different genes annotated to GO Term (* indicates Q-value < 0.05; ** indicates Q-value < 0.01; *** indicates Q-value < 0.001) and y-axis representing GO Term. Different colors represent three GO subclasses: BP, CC, and MF. (**c**) Upregulated genes' KEGG pathway enrichment. (**d**) Downregulated genes' KEGG enrichment analysis, with the x-axis representing the ratio of the number of different genes annotated to the KEGG pathway and added to the total number of genes. Rich factor—the larger the Rich factor, the higher the enrichment. y-axis represents the KEGG pathway. The size of the dot represents the number of genes annotated to the KEGG pathway. The color represents the significance of enrichment—the deeper, the more significant.
